# Sex-specific outcomes in myocardial infarction: a dual-cohort analysis using clinical and real-world data

**DOI:** 10.1007/s00392-025-02627-2

**Published:** 2025-03-20

**Authors:** Johannes Krefting, Christian Graesser, Sophie Novacek, Felix Voll, Aldo Moggio, Nils Krueger, Christian Friess, Marius Schwab, Frank Offenborn, Teresa Trenkwalder, Sebastian Kufner, Erion Xhepa, Michael Joner, Salvatore Cassese, Heribert Schunkert, Gjin Ndrepepa, Adnan Kastrati, Moritz von Scheidt, Thorsten Kessler, Hendrik B. Sager

**Affiliations:** 1 Department of Cardiovascular Diseases, German Heart Centre Munich, School of Medicine and Health, TUM University Hospital, Technical University of Munich, Lazarettstr. 36, 80636 Munich, Germany; 2 German Centre for Cardiovascular Research (DZHK E.V.), Partner Site Munich Heart Alliance, Munich, Germany; 3 Allgemeine Ortskrankenkasse (AOK) Bayern, Munich, Germany

**Keywords:** Sex, Major adverse cardiovascular events, Mortality, Myocardial salvage, ST segment elevation myocardial infarction

## Abstract

**Background:**

Sex-related differences in symptoms, treatment, and outcomes in patients presenting with myocardial infarction have been reported but vary largely between studies. We sought to characterize sex differences in presentation and outcomes of patients with acute ST segment elevation myocardial infarction (STEMI) undergoing primary percutaneous intervention (PPCI).

**Methods and results:**

We included 1206 STEMI patients from a clinical cohort and 35,123 STEMI patients obtained from the German health insurance claims. Women, despite being older and thus having a worse cardiovascular risk profile, had greater myocardial salvage and smaller infarct size than men in all patients (median with [interquartile ranges (25th–75th percentiles), IQR]; salvage index: 0.58 [IQR: 0.32–0.91] in females vs. 0.47 [IQR: 0.23–0.77] in males, *p* < 0.0001; infarct size: 7.0% [IQR: 1.0–22.0%] in females vs. 11.0% [IQR: 3.0–23.0%] of the left ventricle in males, *p* = 0.002). Same results were shown for propensity score matched pairs (*n* = 242) (salvage index: 0.60 [IQR: 0.33–0.91] in females vs. 0.44 [IQR: 0.23–0.70] in males, *p* = 0.0002; infarct size: 7.0% [IQR: 1.0–23.0%] vs. 10% [IQR: 3.0–23.0%] of the left ventricle in males, *p* = 0.042). Furthermore, women showed a lower risk of 5-year mortality, assessed after propensity score matching, in the health insurance cohort (*n* = 19,404) (HR = 0.92 [95% CI 0.87–0.97], *p* = 0.002).

**Conclusions:**

In patients with STEMI, women appear to have better myocardial salvage and smaller infarct size after PPCI and a lower 5-year mortality compared with men, suggesting better ischemic tolerance in female patients.

**Graphical abstract:**

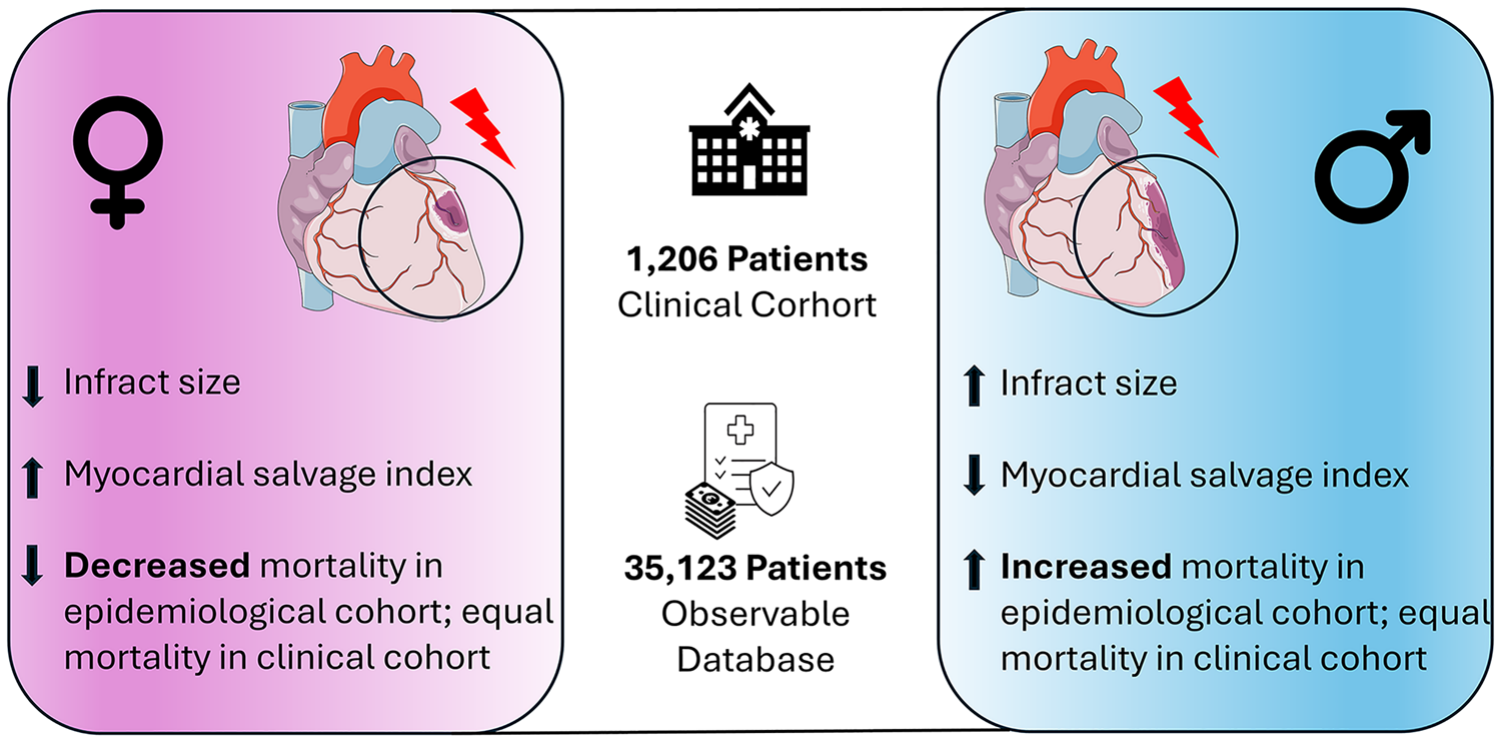

**Supplementary Information:**

The online version contains supplementary material available at 10.1007/s00392-025-02627-2.

## Introduction

Despite recent improvements, cardiovascular disease remains understudied and undertreated in women [1]. For instance, women with ST segment elevation myocardial infarction (STEMI) present later after symptom onset, receive a delayed guideline-recommended therapy, have more MI-related complications and a higher mortality than men with STEMI [2].

While some of these sex disparities may be explained by biological differences between women and men (i.e., estrogens are known to be partially cardioprotective, but levels drop post-menopause), others originate in sex-related sociocultural differences [2]. While women experience MI on average 9 years later than men, MI incidence rises after menopause and then equals that of men [3, 4]. In line, the prevalence of cardiovascular risk factors rises in post-menopausal women. Moreover, the impact of these risk factors on cardiovascular disease progression differs with sex. For instance, smoking and hypertension stronger associate with cardiovascular disease in women than in men [3].

Additionally, substantial differences with respect to presentation, care and outcome in between women and men with MI are reported [5–9]. While men regularly report classical symptoms such as chest pain at the onset of MI, women more often present with angina-atypical symptoms including shortness of breath, nausea and vomiting [10]. These differences often lead to delayed diagnosis and treatment in women. In that light, it was reported that women present later after symptom onset than men with MI [3]. Moreover, most guideline-recommended drugs in the treatment of MI were tested in trials in which women were underrepresented. Moreover, women may respond differently to drug treatment and different drug dosages and treatment durations may be needed in women [11].

Together, most studies, which often do not adjust sufficiently for baseline characteristics, indicate that women with MI receive suboptimal care and have worse outcomes than men [12]. Consequently, higher mortality in women may be attributed to a more unfavorable risk and older age at the time of presentation [13]. Therefore, primary purpose of this study was to explore whether female-intrinsic factors might contribute to severity and recovery process of MI and result in different post-MI outcomes.

What is new? Our data show that in age-matched patients with STEMI, women appear to have better myocardial salvage and smaller infarct sizes after PPCI and a lower 5-year mortality compared to men.

What are the clinical implications? Our data suggest better ischemic tolerance in female STEMI patients which needs further mechanistic investigations and argues for personalized approaches to reduce sex disparities in STEMI care.

## Methods

### Study design

This dual-cohort analysis utilizes a hybrid approach, supplementing clinical data with Real-World Data (RWD) as epidemiological validation. By design, the study represents a retrospective analysis.

Details of the clinical study patients were described before [14–16]. In brief, between January 2002 and December 2007, patients with STEMI undergoing primary percutaneous coronary intervention (PPCI) and serial scintigraphic imaging at two tertiary cardiac care centers (Deutsches Herzzentrum München and Klinikum Rechts der Isar, both Technical University of Munich, Munich, Germany) were included in this study. The diagnosis of STEMI was based on chest pain lasting ≥ 20 min and persistent ST segment elevation ≥ 1 mm in at least two extremity or ≥ 2 mm in at least two chest leads or new onset of left bundle branch block. As reported recently [15], 200 out of 1,406 STEMI patients were excluded because exact time of symptom onset was not clearly documented. Hence, the remaining 1,206 patients were included into this analysis. All patients gave written informed consent for PPCI and imaging procedures.

RWD cohort was derived from observational data collected by the Observational Bavarian Health Insurance Registry (OBSERVABLE). This database is an extensive repository of secondary health claims data provided by the Allgemeine Ortskrankenkasse (AOK) Bayern, a component of the German Statutory Health Insurance system. The OBSERVABLE database includes detailed records from over 1.3 million individuals residing in Bavaria, a state in southern Germany. These individuals are aged 18 years and older and have been diagnosed with atherosclerotic disease between January 1, 2012 and December 31, 2021. The registry contains anonymized patient-level data, which comprises demographic information, diagnoses (coded according to the International Classification of Diseases, 10th Revision, ICD-10), medical procedures, pharmaceutical prescriptions dispensed at pharmacies, and causes of mortality.

For our study, we identified a cohort based on the recorded diagnosis of STEMI, followed by any type of revascularization within 24 h. The cohort was monitored over a five-year period starting from the date of the STEMI to assess clinical outcomes comprehensively. To ensure an adequate assessment of baseline covariates, only those individuals with a minimum of 12 months of continuous enrollment in the database prior to the index revascularization procedure were included. The data covered all levels of care, from tertiary hospitals to outpatient services, thus reflecting a broad spectrum of the healthcare landscape relevant to cardiology. This robust dataset is particularly suited for analyzing clinical outcomes using a mixed-data approach. All used codes can be found in Supplementary Table S1.

#### Ethical considerations and study conduct

The study protocol was approved by the institutional ethics committee (454/21 S-KH for the first and 2019-50-S-SR for the second cohort) and conforms to the Declaration of Helsinki.

### Angiography and PPCI

For the clinical cohort, angiography data were available. The culprit lesion in the infarct-related artery was identified during coronary angiography, based on several angiographic features including the presence of acute occlusion, intraluminal filling defects (or thrombus), ulcerated plaques with contrast-filled pockets protruding into the plaque with or without delayed contrast wash-out, extraluminal contrast, dissection or intraluminal flaps. Coronary artery disease in non-culprit lesions was defined as coronary stenosis of at least 50% lumen obstruction [15]. Left ventricular ejection fraction (LV-EF) on admission (baseline) and after six months was measured on left ventricular angiograms using the area–length method [15]. For periprocedural anticoagulation, unfractionated heparin was administered [15]. The anti-thrombotic regime included an initial loading dose of 600 mg clopidogrel, and aspirin 325 to 500 mg aditionally [15]. Post-procedure, patients received 150 mg clopidogrel until discharge, not exceeding 3 days, mostly followed by maintenance dose of 75 mg/day for at least 1 month. Along with clopidogrel, aspirin 200 mg/day was given indefinitely [15]. Few patients received ticlopidine (250 mg twice/day). In patients presenting with new onset of atrial fibrillation, either aspirin and clopidogrel or aspirin and ticlopidine was combined with phenprocoumon [14].

### Measurement of myocardial area at risk and final infarct size using SPECT

In the clinical cohort, 99mTc-sestamibi single-photon emission computed tomography (SPECT) imaging studies were performed as described previously [14–16]. SPECT imaging was performed twice in each patient at pre-defined time points. For the first measurement*,* 99mTc-sestamibi (27 mCi (1000 MBq)) was injected intravenously prior to PPCI. This was followed by imaging 6–8 h afterward to assess the perfusion defect and by that estimate the myocardial area at risk. During a second measurement*,* 99mTc-sestamibi was injected intravenously again between 7 and 14 days after the PPCI, followed by imaging 6–8 h afterward. Perfusion defects were defined as less than 50% uptake of 99mTc-sestamibi and were expressed as percentage of the left ventricle [14–16]. The myocardial salvage index (i.e., relative salvage) represents the proportion of initial myocardial area at risk salvaged by reperfusion therapy and was calculated as initial myocardial area at risk minus final infarct size divided by initial myocardial area at risk [14, 15]. All measurements were performed by investigators unaware of the clinical or angiographic data of the patients [14–16].

### Laboratory data, medical history and definitions

In the clinical cohort, laboratory measurements were routinely performed daily at the institute of laboratory medicine of our hospital, and data were extracted from patients’ charts up to ten days post-admission. As enzymatic estimate of infarct size creatine kinase myocardial band (CK-MB) and troponin T were measured daily. Peak levels were defined as the highest value obtained during hospitalization. Renal function was evaluated by calculating the creatinine clearance using to the Cockroft–Gault formula.

### Study outcomes and follow-up

The primary outcome of the study was the five-year all-cause mortality rate. For both study groups, major adverse cardiovascular events (MACE), a composite endpoint of acute myocardial infarction, stroke and all-cause death, were evaluated as secondary outcomes. Additionally, within the clinical cohort alone, several specific parameters were assessed: the infarct size, the maximum levels of troponin, the myocardial salvage index, and the LV-EF. These measurements were taken at the time of admission and then again at a six-month follow-up.

As a standard practice in our institutions at the time of patient’s recruitment, six months after the index procedure a repeat coronary angiography was scheduled. The six-month angiograms were used for the assessment of the LV-EF at this time point. For the clinical cohort, the follow-up information was obtained by staff members who were not aware of the clinical data via phone calls 30 days after PCI, 1 year after PCI, and yearly thereafter [14, 15]. Data on mortality were obtained from hospital records, death certificates, or phone contact with patients’ relatives or referring physicians [14, 15].

### Statistical analysis

Continuous data were presented as mean ± standard deviation (SD) or median with interquartile ranges (25th–75th percentiles) [IQR] and 10th–90th percentiles, depending on the normality of the distribution assessed by Shapiro–Wilk test for normality. These data were analyzed using either Independent Samples t-Test or the Mann–Whitney U test. Discrete variables were expressed as proportions (percentages) and analyzed using the chi-square test. A two-sided P value of less than 0.05 was considered statistically significant. We employed the standardized mean difference (SMD) to compare baseline characteristics between male and female within our epidemiological cohort. By convention, a SMD within ± 0.1 was used to define the achievement of a good between-group balance.

To control for potential confounders between female and male participants, we implemented a nearest-neighbor caliper matching without replacement, based on the log-odds of the propensity score (PSM). The propensity scores were calculated using similar pre-exposure covariates, which encompassed baseline demographics and various cardiovascular risk factors present in both the clinical and RWD cohorts (Supplementary Table S2 + S3), thus rendering the groups comparable. The caliper width was set to 20% of the standard deviation of the propensity log-odds. Survival outcomes for the matched cohorts were visualized using Kaplan–Meier survival curves and plotted over a five-year period. Hazard ratios were calculated using Cox proportional hazards models, including variables, such as follow-up times, censoring indicators, and the allocated treatment group, with sex as the primary predictor.

For statistical analysis and data visualization, we utilized Python version 3.10.9, R-Studio version 4.1.2, IBM SPSS Statistics version 29, and GraphPad Prism 9.

## Results

We sought to characterize sex differences in presentation and outcomes in patients with acute MI. Analyses were conducted on datasets from two cohorts: a first clinical cohort comprises 1,206 acute STEMI patients treated with PPCI at two tertiary cardiac centers [14–16] and a second insurance cohort with 35,123 acute STEMI patients obtained from health insurance datasets as contemporary epidemiological validation for clinical outcomes.

**Fig. 1:**
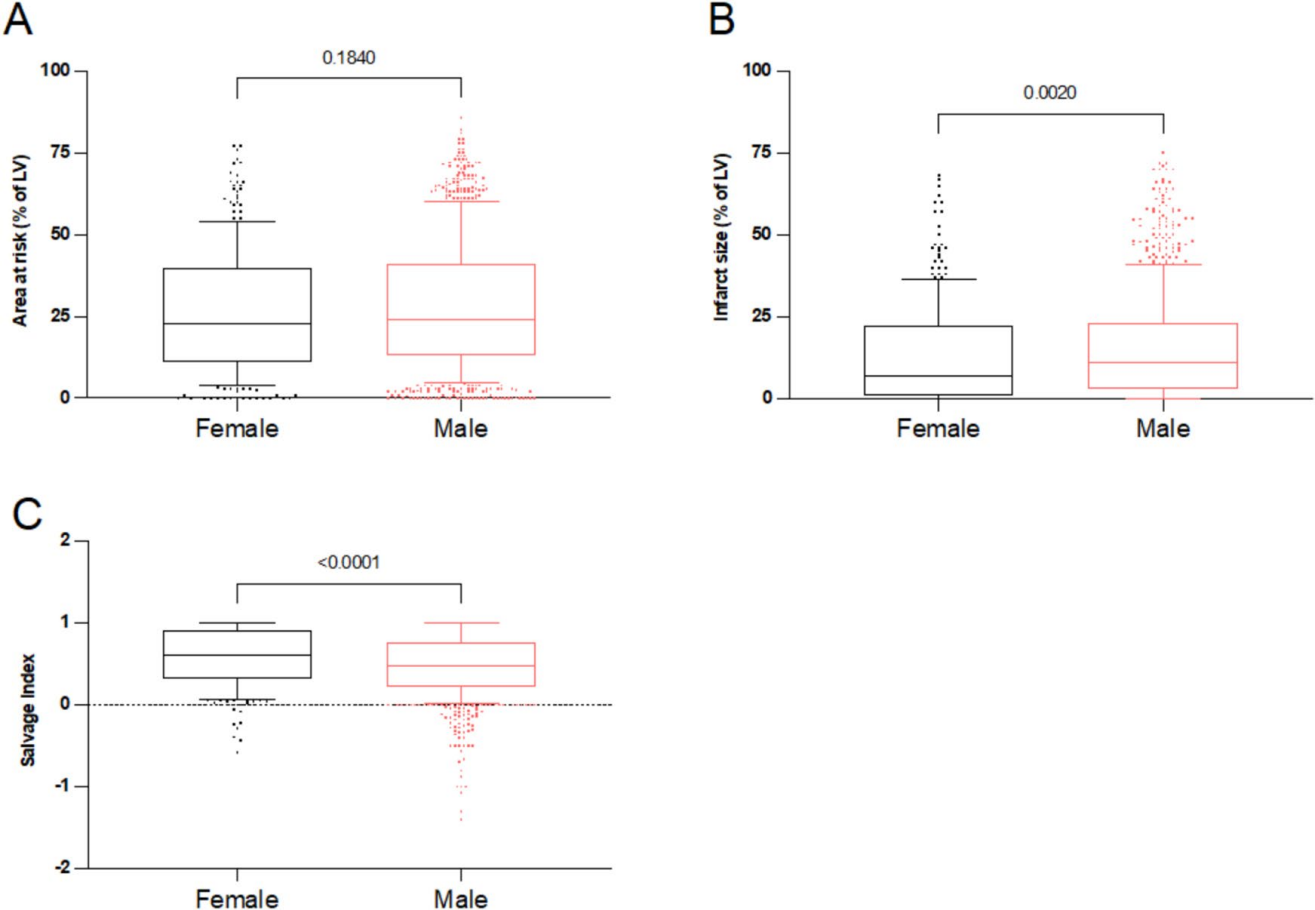
Scintigraphic data in women and men in the clinical cohort prior to propensity score matching. **A** Initial myocardial area at risk (% of the left ventricle) assessed by a first scintigraphic imaging prior to primary percutaneous coronary intervention (PPCI). **B** Final infarct size assessed by a second scintigraphic imaging 7–14 days after PPCI (% of the left ventricle). **C** Myocardial salvage index. Data are presented as median with 25th–75th percentiles (boxes), 10th–90th percentiles (whiskers) and values outside the given percentiles (dots)

**Fig. 2:**
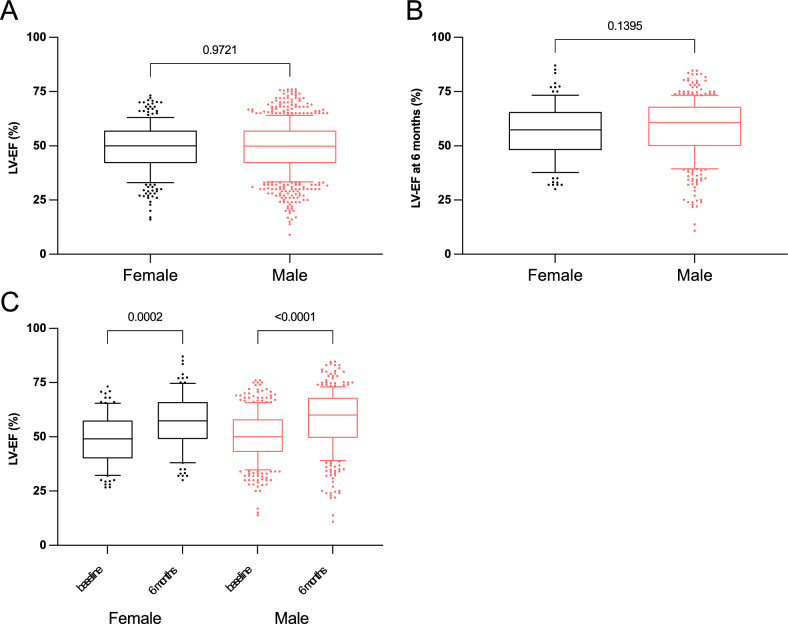
Left ventricular ejection fraction (LV-EF) data in women and men in the clinical cohort prior to propensity score matching. **A** LV-EF at baseline, **B** LV-EF at 6 months after STEMI and **C** comparison between baseline and 6 months between female and male patients. Data are presented as median with 25th–75th percentiles (boxes), 10th–90th percentiles (whiskers) and values outside the given percentiles (dots)

We started analyzing the first cohort of STEMI patients (*n* = 1206) of which 286 (23.7%) were female and 920 (76.3%) were male. The patients’ characteristics are shown in Table 1. On average, female patients were more than nine years older than male patients. The prevalence of type 2 diabetes, hypertension and renal failure was higher, whereas the prevalence of smoking and obesity was lower in female patients. Female patients presented at the emergency department approximately 1 h later after symptom onset than male patients: mean ± SD (7.41 ± 5.97 h in females vs. 6.47 ± 5.98 h in males, *p* < 0.001, Supplementary Fig. S1A). The door-to-balloon time was similar between the groups (1.42 ± 0.79 h in females vs. 1.41 ± 0.84 h in males, Supplementary Fig. S1B). The initial myocardial area at risk before PPCI was not different (23.0% [IQR: 11.0–39.5%] in females vs. 24.0% [IQR: 13.0–41.0%] of the left ventricle in males) although women presented later after symptom onset (Fig. 1A). The infarct size as estimated by a second SPECT imaging was significantly smaller in females (7.0% [IQR: 1.0–22.0%] in females vs. 11.0% [IQR: 3.0–23.0%] in males, *p* = 0.002, Fig. 1B). Myocardial salvage index was higher in females (0.58 [IQR: 0.32–0.91] in females vs. 0.47 [IQR: 0.23–0.77] in males, *p* < 0.0001, Fig. 1C). In line with the scintigraphic assessment of the infarct size, peak creatine kinase myocardial band (CK-MB) levels were lower in females (101.50 U/L [IQR: 40.00–205.25 U/L) in females vs. 112.50 U/L [IQR: 54.22–230.25 U/L] in males, *p* = 0.047, Supplementary Fig. S2A), whereas peak troponin T levels were not different (3.16 ng/ml [IQR: 1.44–7.19 ng/ml] in females vs. 3.77 ng/ml [IQR: 1.61–7.55 ng/ml] in males, *p* = 0.149, Supplementary Fig. S2B). LV-EF data were available in 270 female patients (94.4%) and in 877 male patients (95.3%). LV-EF did not differ between female and male patients at baseline (50.0% [IQR: 42.0–57.0%] in females vs. 49.9% [IQR: 42.0–57.0%] in males, *p* = 0.972, Fig. 2A) and at 6 months of follow-up (57.4% [IQR: 48.2—65.5%] in females vs. 60.8% [IQR: 49.9–68.0%] in males, *p* = 0.139, Fig. 2B). A substantial LV-EF recovery was present in both female and male patients (Fig. 2C). Together, even though women presented almost one hour later after symptom onset, females showed better myocardial salvage and smaller infarct sizes than male patients.

**Table 1:** Baseline characteristics of clinical cohort before (left) and after (right) propensity score matching

Baselines	Male (*n* = 920) unmatched	Female (*n* = 286) unmatched	*p*-value	Male (*n* = 242) matched	Female (*n* = 242) matched	*p*-value
Age	59.53 (± 12.16)	68.98 (± 12.90)	< 0.001	67.09 (± 12.30)	66.59 (± 12.31)	0.750
BMI	26.87 (± 3.65)	26.11 (± 4.64)	< 0.001	25.99 (± 3.50)	26.02 (± 4.71)	0.526
Diabetes	157 (17.07%)	72 (25.17%)	0.003	58 (23.97%)	53 (21.90%)	0.665
Hypercholesterolemia	495 (53.80%)	155 (54.20%)	0.962	130 (53.72%)	132 (54.55%)	0.927
Hypertension	623 (67.72%)	223 (77.97%)	0.001	178 (73.55%)	182 (75.21%)	0.755
Smoking	429 (46.63%)	88 (30.77%)	< 0.001	86 (35.54%)	82 (33.88%)	0.775
Family history of CAD	360 (39.13%)	120 (41.96%)	0.433	103 (42.56%)	102 (42.15%)	0.999
An. MI	121 (13.15%)	30 (10.49%)	0.277	29 (11.98%)	30 (12.40%)	0.999
An. ACVB	32 (3.48%)	7 (2.45%)	0.503	8 (3.31%)	7 (2.89%)	0.999
GFR	94.08 (± 32.32)	66.58 (± 30.51)	< 0.001	72.20 (± 29.05)	71.38 (± 30.49)	0.524
CAD	1.99 (± 0.83)	1.95 (± 0.83)	0.524	2.15 (± 0.84)	1.93 (± 0.83)	0.003
Killip Class	1.34 (± 0.69)	1.41 (± 0.71)	0.034	1.45 (± 0.80)	1.38 (± 0.68)	0.443
TIMI pre-intervention	1.13 (± 1.35)	1.21 (± 1.19)	0.252	1.32 (± 1.73)	1.21 (± 1.20)	0.822
TIMI post-intervention	2.79 (± 0.57)	2.83 (± 0.54)	0.297	2.81 (± 0.56)	2.86 (± 0.50)	0.195
Time to admission	6.47 (± 5.98)	7.41 (± 5.97)	< 0.001	6.82 (± 5.86)	7.28 (± 5.85)	0.203
Time to intervention	1.41 (± 0.84)	1.42 (± 0.79)	0.589	1.37 (± 0.82)	1.40 (± 0.80)	0.891
PTCA	BMS: 581 (63%) DES: 214 (23%) PTCA: 125 (14%)	BMS: 176 (61.5%) DES: 73 (24.5%) PTCA: 37 (13%)	0.730	BMS: 165 (68%) DES: 46 (19%) PTCA: 31 (13%)	BMS: 153 (63%) DES: 57 (24%) PTCA: 32 (13%)	0.440
Vessel	LAD: 404 (44%) RCA: 332 (36%) LCx: 163 (18%) ACVB: 21 (2%)	LAD: 139 (49%) RCA: 107 (37%) LCx: 39 (14%) ACVB: 1 (< 1%)	0.058	LAD: 101 (42%) RCA: 93 (38%) LCx: 44 (18%) ACVB: 4 (2%)	LAD: 122 (51%) RCA: 88 (36%) LCx: 31 (13%) ACVB: 1 (< 1%)	0.175

Data are presented as mean ± SD

BMI: Body Mass Index; CAD: Coronary artery disease; MI: Myocardial infarction; CABG: coronary artery bypass grafting; GFR: Glomerular filtration rate; TIMI: Thrombolysis in Myocardial Infarction; PCI: percutaneous coronary intervention; SD: standard deviation

**Fig. 3:**
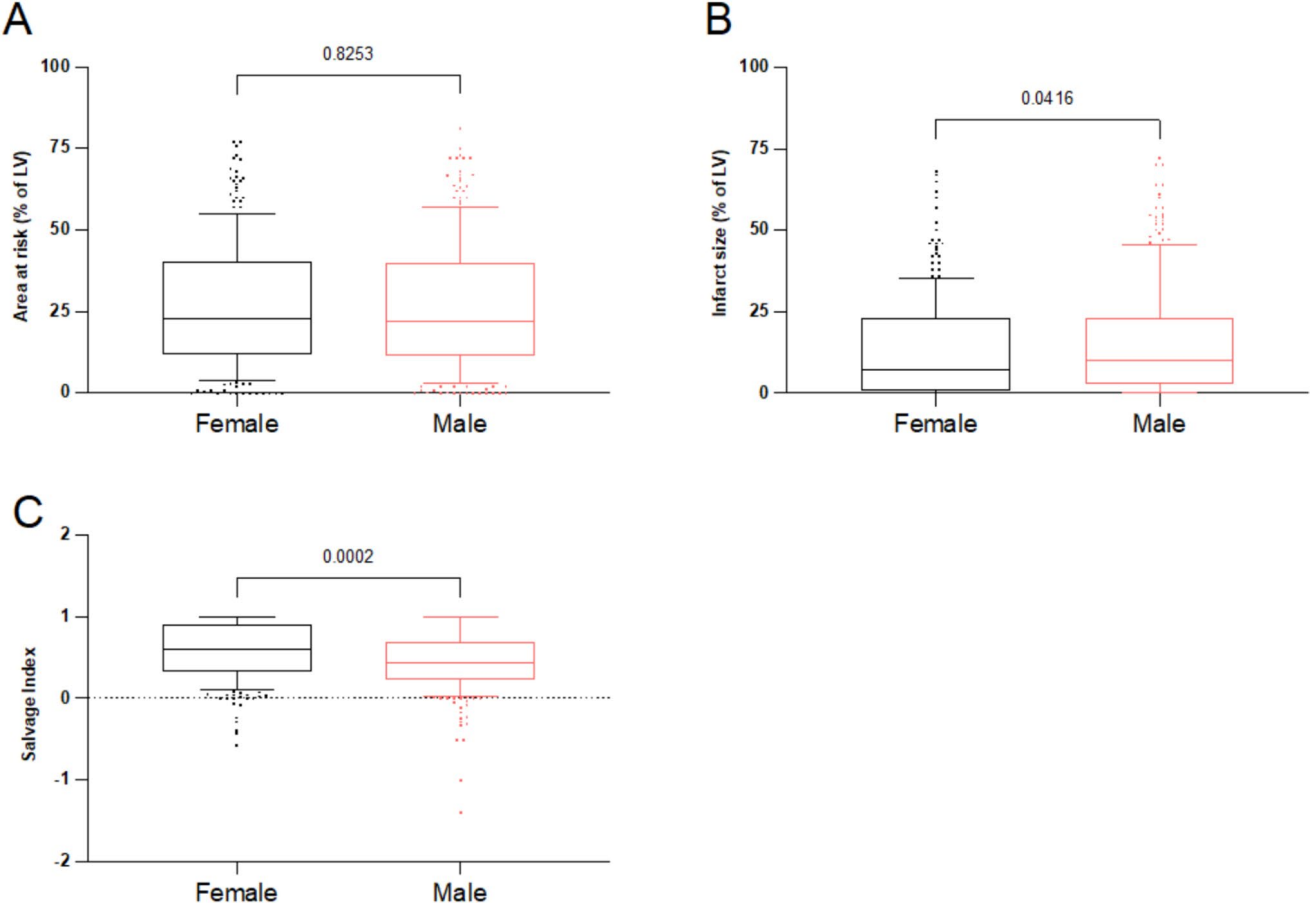
Scintigraphic data in women and men in the clinical cohort after propensity score matching. **A** Initial myocardial area at risk (% of the left ventricle) assessed by a first scintigraphic imaging prior to primary percutaneous coronary intervention (PPCI). **B** Final infarct size assessed by a second scintigraphic imaging 7–14 days after PPCI (% of the left ventricle). **C** Myocardial salvage index. Data are presented as median with 25th—75th percentiles (boxes), 10th—90th percentiles (whiskers) and values outside the given percentiles (dots)

**Fig. 4:**
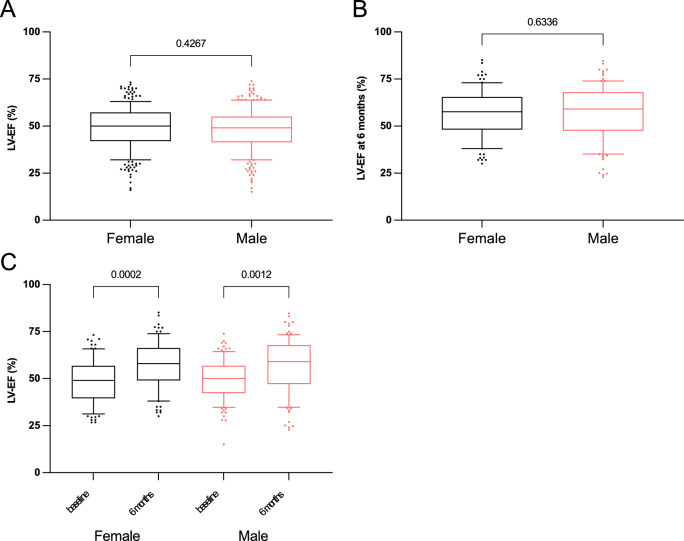
Left ventricular ejection fraction (LV-EF) data in women and men in the clinical cohort after propensity score matching. **A** LV-EF at baseline, **B** LV-EF at 6 months after STEMI and **C** comparison between baseline and 6 months between female and male patients. Data are presented as median with 25th–75th percentiles (boxes), 10th–90th percentiles (whiskers) and values outside the given percentiles (dots)

Considering differences in baseline characteristics between female and male patients, propensity score matching was performed (242 matched pairs). The baseline characteristics are shown in Table 1. In the matched groups, the time-to-admission and the door-to-balloon time did not differ between female and male patients (Supplementary Fig. S3). While the area at risk of the left ventricle was the same between females and males (23.0% [IQR: 12.0–40.0%] in females vs. 21.9% [IQR: 11.8–39.8%] in males, *p* = 0.825, Fig. 3A), the final infarct size was smaller in females (7.3% [IQR: 1.0–23.0%] in females vs. 10.1% [IQR: 3.0–23.0%] in males, *p* = 0.041, Fig. 3B). Therefore, myocardial salvage index was higher in female patients (0.60 [IQR: 0.33–0.91] in females vs. 0.44 [IQR: 0.23–0.70] in males, *p* = 0.0002, Fig. 3C). Peak CK-MB level did not differ significantly between patients (95.0 U/L [IQR: 38.0–194.5 U/L] in females vs. 94.0 U/L [IQR: 49.0—222.0 U/L] in males, *p* = 0.339), whereas peak Troponin T levels were lower in female patients (3.02 ng/ml [IQR: 1.42—6.92 ng/ml] in females vs. 4.58 ng/ml [IQR: 1.61–8.81 ng/ml] in males, *p* = 0.031, Supplementary Fig. S4). Angiographic LV-EF measurements were available in 229 (94.6%) of the female and in 225 (93.0%) of the male patients. There were no significant differences in the LV-EF between patients at baseline (50.0% [IQR: 42.0–57.0%] in females vs. 49.0% [IQR: 41.7—55.0%] in males, *p* = 0.426, Fig. 4A) and at 6 months thereafter (57.7% [IQR: 48.5—65.2%] in females vs. 59.2% [IQR: 47.9–68.0%] in males, *p* = 0.633, Fig. 4B). Significant recovery was present in both groups (Fig. 4C). Thus, in propensity matched pairs, female patients with STEMI showed better myocardial salvage and smaller infarcts compared with male patients.

**Fig. 5:**
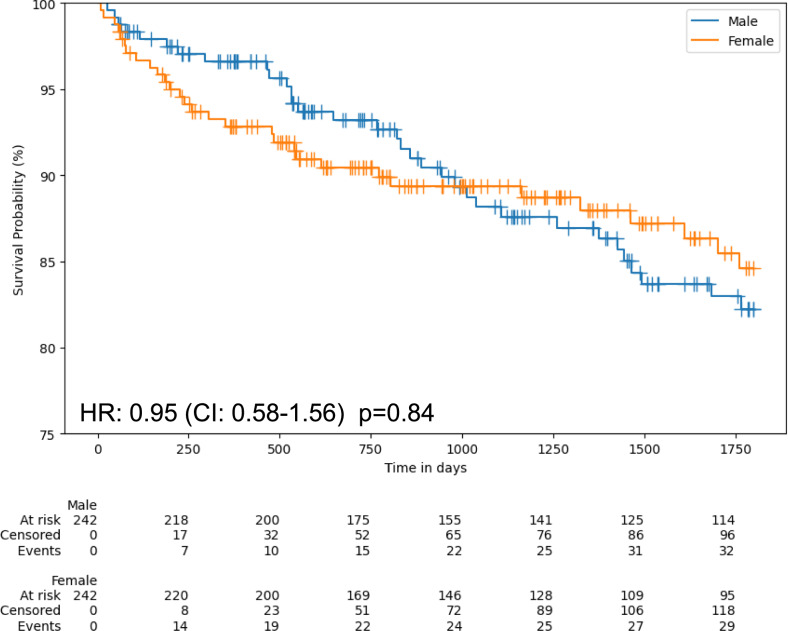
Kaplan–Meier curves of five-year all-cause mortality between female and male patients in the clinical cohort after propensity score matching. CI = confidence interval; HR = hazard ratio

We next assessed outcomes in this cohort. The median follow-up was 1,396 days. The primary endpoint (death of any cause at five years) occurred in 102 (8.5%) patients: 37 female and 65 male patients had died (Kaplan–Meier estimates of five-year mortality: 12.9% in females and 7.1% in males, HR = 2.00 [95% CI: 1.34–2.98], *p* < 0.001, Supplementary Fig. S5). At five years, MACE occurred in 374 patients: 101 events in female and 273 events male patients (Kaplan–Meier estimates of five-year MACE: 35.3% in females and 29.7% in males, HR = 1.26 [95% CI: 1.01–1.59], *p* = 0.04, Supplementary Fig. S6). Female patients showed a higher five-year all-cause mortality and reduced five-year event-free survival from MACE in comparison to male patients. However, these differences disappeared after propensity score matching. Among propensity matched pairs, 61 patients had died at five years (29 female and 32 male patients, Kaplan–Meier estimates of five-year mortality: 12.0% in females and 13.2% in males, HR = 0.95 [95% CI: 0.58–1.56], *p* = 0.84, Fig. 5). MACE occurred in 171 patients (87 events in female and 84 events male patients, Kaplan–Meier estimates of five-year MACE: 36.0% in females and 34.7% in males, HR = 1.05 [95% CI: 0.78–1.41], *p* = 0.75, Supplementary Fig. S7).

The health insurance dataset included 35,123 acute STEMI patients: 10,498 (30.0%) female and 24,625 (70%) male patients. The patients’ characteristics are shown in Table 2 and Supplementary Fig. S8. Female patients were on average 8 years older at the time of admission for STEMI than male patients. Female patients showed a higher five-year all-cause mortality compared with male patients. The primary endpoint (5-year incidence of death of any cause) occurred in 8,135 patients: 3135 female and 5,000 male patients (Kaplan–Meier estimates of five-year mortality: 30.0% in females and 20.3% in males, HR = 1.56 [95% CI: 1.49–1.63], *p* < 0.001, Supplementary Fig. S9). MACE occurred in 11,466 patients: 4,012 events in female and 7,454 events in male patients (Kaplan–Meier estimates of five-year MACE: 38.2% in females and 30.3% in males, HR = 1.33 [95% CI: 1.28—1.38], *p* < 0.001, Supplementary Fig. S10).

**Table 2:** Baseline characteristics of health insurance cohort before (left) and after (right) propensity score matching

Baselines	Male (*n* = 24,625) unmatched	Female (*n* = 10,498) unmatched	SMD	Male (*n* = 9,702) matched	Female (*n* = 9,702) matched	SMD
Age	63.3 (12.5)	71.6 (12.9)	0.648	70.3 (12.3)	70.3 (12.6)	0.003
Obesity	5506 (22.4%)	2850 (27.1%)	0.111	2624 (27.0%)	2544 (26.2%)	− 0.019
Diabetes	8012 (32.5%)	4312 (41.1%)	0.178	3890 (40.1%)	3887 (40.1%)	− 0.001
Insulin-Use	1922 (7.8%)	1205 (11.5%)	0.125	1040 (10.7%)	1038 (10.7%)	− 0.001
Hyperlipidemia	18,141 (73.7%)	7847 (74.7%)	0.025	7260 (74.8%)	7276 (75.0%)	0.004
Hypertension	19,899 (80.8%)	9349 (89.1%)	0.232	8247 (85.0%)	8317 (85.7%)	0.020
Smoking	6549 (26.6%)	1874 (17.9%)	− 0.211	1870 (19.3%)	1858 (19.2%)	− 0.003
Previous MI	4436 (18.0%)	1411 (13.4%)	− 0.126	1367 (14.1%)	1393 (14.4%)	0.008
Previous CABG	19 (0.1%)	6 (0.1%)	− 0.008	8 (0.1%)	6 (0.1%)	− 0.008
CKD	4520 (18.4%)	3043 (29.0%)	0.252	2668 (27.5%)	2596 (26.8%)	− 0.017
AFib	2517 (10.2%)	1561 (14.9%)	0.141	1383 (14.3%)	1373 (14.2%)	− 0.003
1 Vessel CVD	7546 (30.6%)	3602 (34.3%)	0.078	2541 (26.2%)	3376 (34.8%)	0.188
2 Vessel CVD	7639 (31.0%)	3140 (29.9%)	− 0.024	2913 (30.0%)	2935 (30.3%)	0.005
3 Vessel CVD	10,023 (40.7%)	3812 (36.3%)	− 0.090	4535 (46.7%)	3463 (35.7%)	− 0.226
LM Vessel CVD	1714 (7.0%)	641 (6.1%)	− 0.035	826 (8.5%)	590 (6.1%)	− 0.094
Cardiogenic Shock	3025 (12.3%)	1567 (14.9%)	0.077	1431 (14.7%)	1377 (14.2%)	− 0.016

Plus-minus values are means ± SD. Values are provided as *n* (%). Differences are shown as standardized mean difference (SMD)

CKD: Chronical kidney disease; CAD: Coronary artery disease; MI: Myocardial infarction; CHF: Chronical heart failure; PAD: peripheral artery disease; PCI: percutaneous coronary intervention; CABG: coronary artery bypass grafting; COPD: Chronic obstructive pulmonary disease; SD: standard deviation

**Fig. 6:**
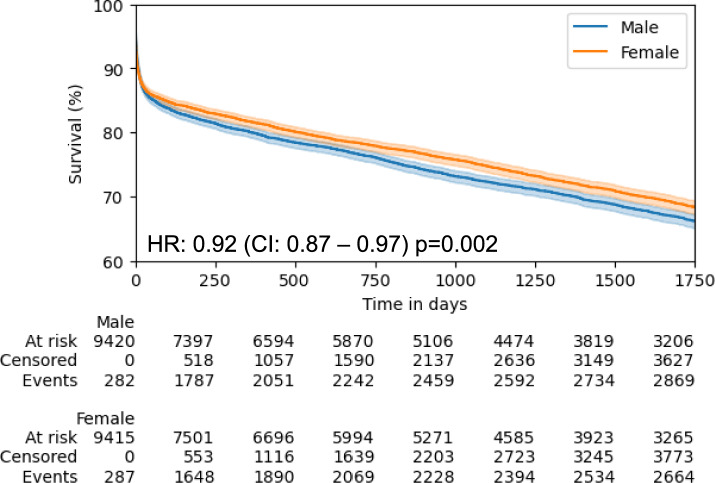
Kaplan–Meier curves of five-year all-cause mortality in propensity score matched women and men in the health insurance cohort after propensity score matching. CI = confidence interval; HR = hazard ratio

Since female and male patients differed significantly with respect to age and comorbidities, we again performed a propensity score matching to adjust for differences in baseline characteristics (9,702 female/male pairs). The baseline characteristics are shown in Table 2 and Supplementary Fig. S11. Females showed reduced mortality and higher event-free survival from MACE indicating a protective effect in female patients. Death of any cause at five years occurred in 5,533 matched patients: 2,664 female and 2,869 male patients had died (Kaplan–Meier estimates of five-year mortality: 44.9% in females and 47.2% in males, HR = 0.92 [95% CI: 0.87–0.97], *p* = 0.002, Fig. 6). MACE occurred in 7,293 matched patients: 3,487 events in female and 3,806 events male patients (Kaplan–Meier estimates of five-year MACE: 27.5% in females and 29.6% in males, HR = 0.90 [95% CI: 0.86—0.94], *p* < 0.001, Supplementary Fig. S12). When we compared females and males with acute MI complicated by cardiogenic shock (AMICS), we did not find differences in survival after propensity score matching (Supplementary Fig. S13 + S14). Together, women with STEMI showed reduced mortality and higher event-free survival from MACE than matched male patients in this large-scale dataset.

## Discussion

STEMI care is unequally provided to women and men leading to profound differences in rates of adverse events and mortality [12]. However, reports are controversial and large-scale data on this topic are scare. We report that, after adjusting for age and risk factor distribution, female patients with STEMI (i) showed beneficial myocardial salvage and smaller infarct sizes, and (ii) were found to have decreased mortality and cardiac event rates in comparison to male MI patients.

Our study revealed that women with STEMI presented at an older age in comparison to men. In both cohorts, women were on average 9–10 years older than males with STEMI which is exactly in line with other studies [12]. With increasing age, the prevalence of cardiovascular risk factors, which significantly contribute to the development and course of MI, was also different between females and males with MI. Women more often suffered from diabetes and hypertension. This finding is in line with other studies which also showed that women with STEMI presented with a differential burden of cardiovascular co-morbidities [12]. There are several possibilities which might explain why women acquire cardiovascular disease later than men. Men seem to suffer from MI earlier because they have higher risk factor burden at a younger age than women [17]. Women may be partially protected by higher estrogen levels which however decrease with menopause. This may explain why the incidence of coronary heart disease is markedly lower among women prior to the age of 50 years after which time the incidence increases and approaches that of men [17].

As in previous reports [1, 3], our findings showed that women reported on average one hour later after the onset of MI symptoms at emergency departments than men. This delay may adversely affect women since guidelines recommend prompt revascularization in the STEMI setting [18]. Reasons for this delayed presentation may be either patient-centered or medical professional-centered [1]. Females experience classical angina symptoms less often in MI than men and may hence dismiss and misclassify their symptoms. Moreover, emergency medical teams tending to women with MI may also misinterpret symptoms more often. This may contribute to the fact that women are known to receive guideline-recommended anti-thrombotic and anti-anginal pre-hospital treatment less often [1]. After adjusting for age, however, the difference in time-to-admission between the groups lost statistical significance indicating that specifically older patients report later after MI symptom onset.

As in pre-hospital management, women are also reported to receive less and delayed guideline-recommended in-hospital MI treatment than males. Women with STEMI are known to have longer door-to-balloon times [12]. However, in our 1,206 patients with MI, timely reperfusion was equally delivered to women as to men as the door-to-balloon time was unchanged between groups. As in the pre-hospital situation, non-classical MI symptoms that women present more often may cause these reported delays. However, disparities in in-hospital treatment can be improved by implementation of a comprehensive STEMI protocol [12]. Beyond reperfusion therapy, women may also respond differently to post-MI medical therapy. In that light, it was described that men with acute coronary syndromes benefit more from prasugrel therapy than woman [11]. For most post-MI-applied drugs, there is higher evidence for men since women are generally underrepresented in many randomized controlled trials [19].

Despite the fact that women presented later after symptom onset, we surprisingly found that myocardial salvage was increased and infarcted sizes were smaller in women. Findings from both the scintigraphic and enzymatic assessment of the infarct size were in line. Moreover, these findings were consistent in both unadjusted and adjusted (for age and risk factors) analyses. These results may indicate a better ischemic tolerance in women as proposed by another study [20]. However, smaller infarct sizes did not lead to a better preserved left ventricular function in female patients, although assessment of left ventricular function beyond 6 months and data on heart failure medication were not available.

With respect to the subsequent outcome after STEMI, a higher mortality was found in women compared with men mostly in unadjusted analyses. Similar findings have been already reported [6, 19]. However, after matching for age and the differences in the prevalence of risk factors between women and men, the sex-related differences in mortality were attenuated. The fact that women showed a better myocardial salvage and smaller infarct sizes may have caused these differences in outcome. The attenuation of the association after adjustment may point out to the impact of more frequent cardiovascular risk factors and co-morbidities among women, which may counteract the beneficial effect of better myocardial salvage in women with STEMI. Our report argues for a better ischemic tolerance in female STEMI patients, a fact known from pre-clinical studies where female mice also show better short- and mid-term survival after MI than age-matched male mice [21]. We can only speculate as to why women tend to tolerate ischemia better than men. First, women benefit from the cardioprotective effects of estrogen, which are well-documented. Second, they may have a microvascular circulation that better adapts to coronary occlusion. Third, studies suggest that women have a more efficient myocardial metabolism, allowing for better energy production during ischemia. Fourth, women may exhibit a more favorable inflammatory response following myocardial infarction, which could limit tissue damage and enhance recovery. Additionally, women might benefit from improved ischemic preconditioning and distinct neural responses [1–3].

The employment of a dual-cohort hybrid analysis, which combines clinical data with secondary health claims data, merges the precision of clinical environments with the expansiveness of real-world settings. This method allowed us to evaluate a clinical cohort of 1,206 STEMI patients and a larger dataset of 35,123 patients from German health insurance claims. Using PSM to adjust for risk factors and balance sex ratios, we effectively compared outcomes and survival rates across both datasets, identifying differences between the cohorts at a detailed diagnostic level within the clinical setting, and understanding outcomes and events within the extensive real-world data cohort.

## Limitations

Our study has several limitations that need be considered when interpreting the findings. First, the analysis was retrospective and derived from an observational study and not from a prospective clinical trial specifically aimed at investigating the association between sex and outcomes in patients with STEMI. As such, the results should be considered primarily hypothesis-generating. Second, the clinical cohort, which included patients enrolled from 2002 to 2007, received anti-platelet therapy (primarily clopidogrel), which is now considered outdated in the setting of STEMI. Furthermore, the loading dose, the maintenance dose, and the duration of clopidogrel therapy reflected institutional practice at the time and no longer align with current guideline recommendations. Similarly, aspirin dosing (200 mg per day indefinitely) was higher than current recommendations and again reflected institutional practice at that time. Consequently, our findings may not directly translate to patients receiving current, more advanced anti-platelet treatments. However, the real-world data cohort includes a more contemporary patient population with STEMI (2013 to 2021) reflecting current practices and medications. The use of observational health claims data presents its own challenges, especially as it often lacks specific clinical details such as laboratory values. While ICD-10 codes offer a standardized way to classify medical diagnoses, the accuracy of these codes heavily relies on the precision and judgment of the coding physicians. Fortunately, this is less of an issue in our study, which focuses on STEMI, a diagnosis typically clear-cut and supported by definitive ECG signs and test results. Nonetheless, this remains a point of consideration for the overall reliability of the data used in our analysis. Further, relying on a German health claims database closely mirrors the demographics of a Western European cohort, which may limit the broader applicability of our results to different ethnicities and healthcare systems. Finally, data on estrogen replacement therapy which may have impacted the results were not available in female MI patients.

## Conclusion

In patients with STEMI, women appear to have better myocardial salvage and smaller infarct sizes after PPCI and a lower 5-year mortality compared to men. Our data suggest better ischemic tolerance in female STEMI patients which needs further mechanistic investigations and argues for personalized approaches to reduce sex disparities in STEMI care.

## Supplementary Information

Below is the link to the electronic supplementary material.

Supplementary file 1 (DOCX 2056 kb)

## Data Availability

The data supporting the findings of this study are available from the corresponding author upon reasonable request.

## Abbreviations

MACE: Major adverse cardiovascular events
PPCI: Primary percutaneous coronary intervention
WHO: World Health Organization
MI: Myocardial infarction
STEMI: ST segment elevation myocardial infarction
RWD: Real-World Data
OBSERVABLE: Observational bavarian health insurance registry
AOK: Allgemeine Ortskrankenkasse
ICD: International classification of diseases
SPECT: Single-photon emission computed tomography
PSM: Propensity score matching
