# Correction: Sex-specific outcomes in myocardial infarction: a dual-cohort analysis using clinical and real-world data

**DOI:** 10.1007/s00392-025-02678-5

**Published:** 2025-05-19

**Authors:** Johannes Krefting, Christian Graesser, Sophie Novacek, Felix Voll, Aldo Moggio, Nils Krueger, Christian Friess, Marius Schwab, Frank Offenborn, Teresa Trenkwalder, Sebastian Kufner, Erion Xhepa, Michael Joner, Salvatore Cassese, Heribert Schunkert, Gjin Ndrepepa, Adnan Kastrati, Moritz von Scheidt, Thorsten Kessler, Hendrik B. Sager

**Affiliations:** 1 Department of Cardiovascular Diseases, German Heart Centre Munich, School of Medicine and Health, TUM University Hospital, Technical University of Munich, Lazarettstr. 36, 80636 Munich, Germany; 2 German Centre for Cardiovascular Research (DZHK E.V.), Partner Site Munich Heart Alliance, Munich, Germany; 3 Allgemeine Ortskrankenkasse (AOK) Bayern, Munich, Germany

**Correction: Clinical Research in Cardiology** 10.1007/s00392-025-02627-2

When this article was published, the column headings "Male" and "Female" in Table 2 were reversed. The values themselves were not affected.

The Original Article has been corrected.

