# Supplementary material for: Sex-specific outcomes in myocardial infarction: a dual-cohort analysis using clinical and real-world data
Source: Clin Res Cardiol. 2025 Mar 20;114(12):1692–704. doi: 10.1007/s00392-025-02627-2 (PMC12708805; doi:10.1007/s00392-025-02627-2)
Supplement: Supplementary file 1 — (DOCX 2056 kb) [file 392_2025_2627_MOESM1_ESM.docx]

**Supplementary Figures and Tables**

**Supplementary Fig. S1:** **(A)** Time-to-admission and **(B)** door-to-balloon time intervals in women and men in the clinical cohort prior to propensity score matching. Data are presented as median with 25^th^ - 75^th^ percentiles (boxes), 10^th^ - 90^th^ percentiles (whiskers) and values outside the given percentiles (dots).


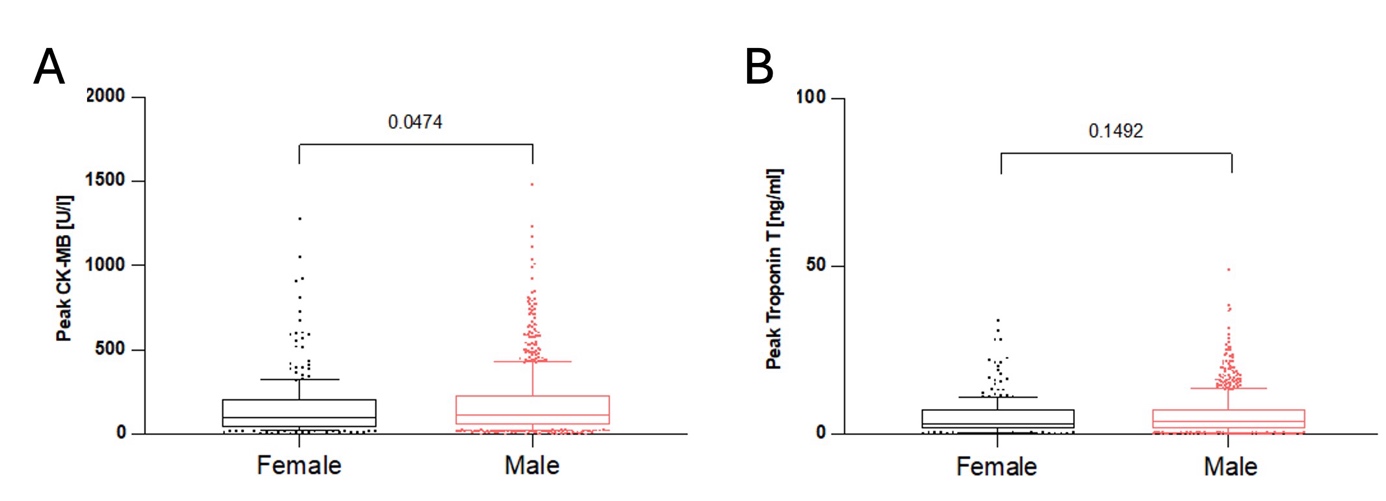


**Supplementary Fig. S2: (A)** Peak blood creatine kinase myocardial band (CK-MB) and **(B)** peak troponin T values in women and men in the clinical cohort prior to propensity score matching. Data are presented as median with 25^th^ - 75^th^ percentiles (boxes), 10^th^ - 90^th^ percentiles (whiskers) and values outside the given percentiles (dots).

**Supplementary Fig. S3:** **(A)** Time-to-admission and **(B)** door-to-balloon time intervals in women and men in the clinical cohort after propensity score matching. Data are presented as median with 25^th^ - 75^th^ percentiles (boxes), 10^th^ - 90^th^ percentiles (whiskers) and values outside the given percentiles (dots).

**Supplementary Fig. S4: (A)** Peak blood creatine kinase myocardial band (CK-MB) and **(B)** peak troponin T values in women and men in the clinical cohort after propensity score matching. Data are presented as median with 25^th^ - 75^th^ percentiles (boxes), 10^th^ - 90^th^ percentiles (whiskers) and values outside the given percentiles (dots).


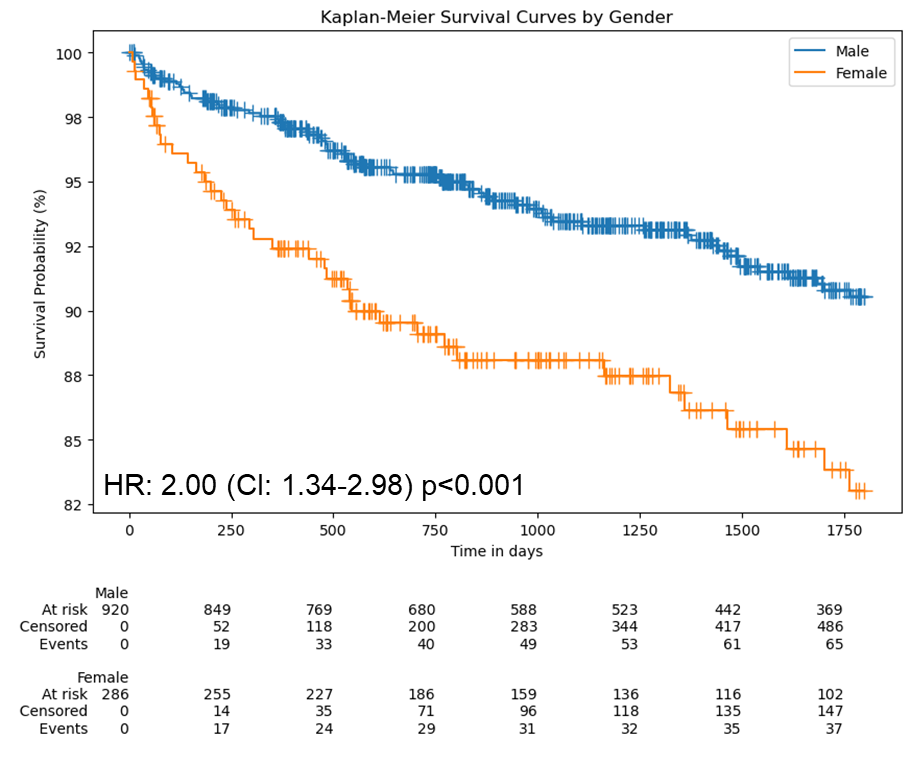


**Supplementary Fig. S5:** Kaplan-Meier curves of five-year all-cause mortality between female and male patients in the clinical cohort prior to propensity score matching. CI=confidence interval; HR=hazard ratio.

**Supplementary Fig. S6:** Five-year event-free survival from major adverse cardiovascular events (MACE), a composite of myocardial infarction, stroke or death, in the clinical cohort prior to propensity score matching. CI=confidence interval; HR=hazard ratio.

**Supplementary Fig. S7:** Five-year event-free survival from major adverse cardiovascular events (MACE), a composite of myocardial infarction, stroke or death, in the clinical cohort after propensity score matching. CI=confidence interval; HR=hazard ratio.


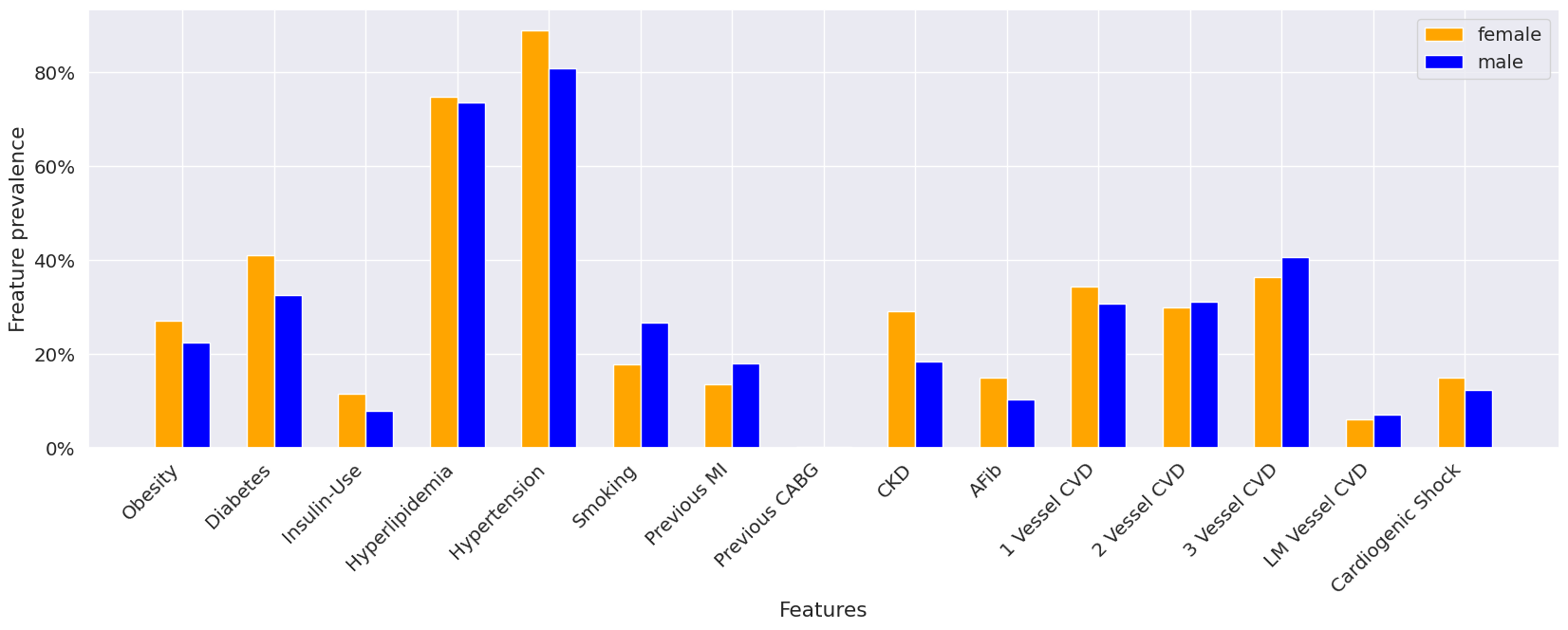


**Supplementary Fig. S8:** Epidemiological and clinical data in the dataset from German health insurance claims prior to propensity score matching. CKD: chronic kidney disease, AFib: atrial fibrillation, CVD: cardiovascular disease, MI: myocardial infarction, CHF: chronic heart failure, PAD: peripheral artery disease, PCI: percutaneous coronary intervention, CABG: coronary artery bypass graft surgery.****

**Supplementary Fig. S9:** Kaplan-Meier curves of five-year all-cause mortality between female and male patients in the health insurance cohort prior to propensity score matching. CI=confidence interval; HR=hazard ratio.

**Supplementary Fig. S10:** Five-year event-free survival from major adverse cardiovascular events (MACE), a composite of myocardial infarction, stroke or death, in the health insurance cohort prior to propensity score matching. CI=confidence interval; HR=hazard ratio.


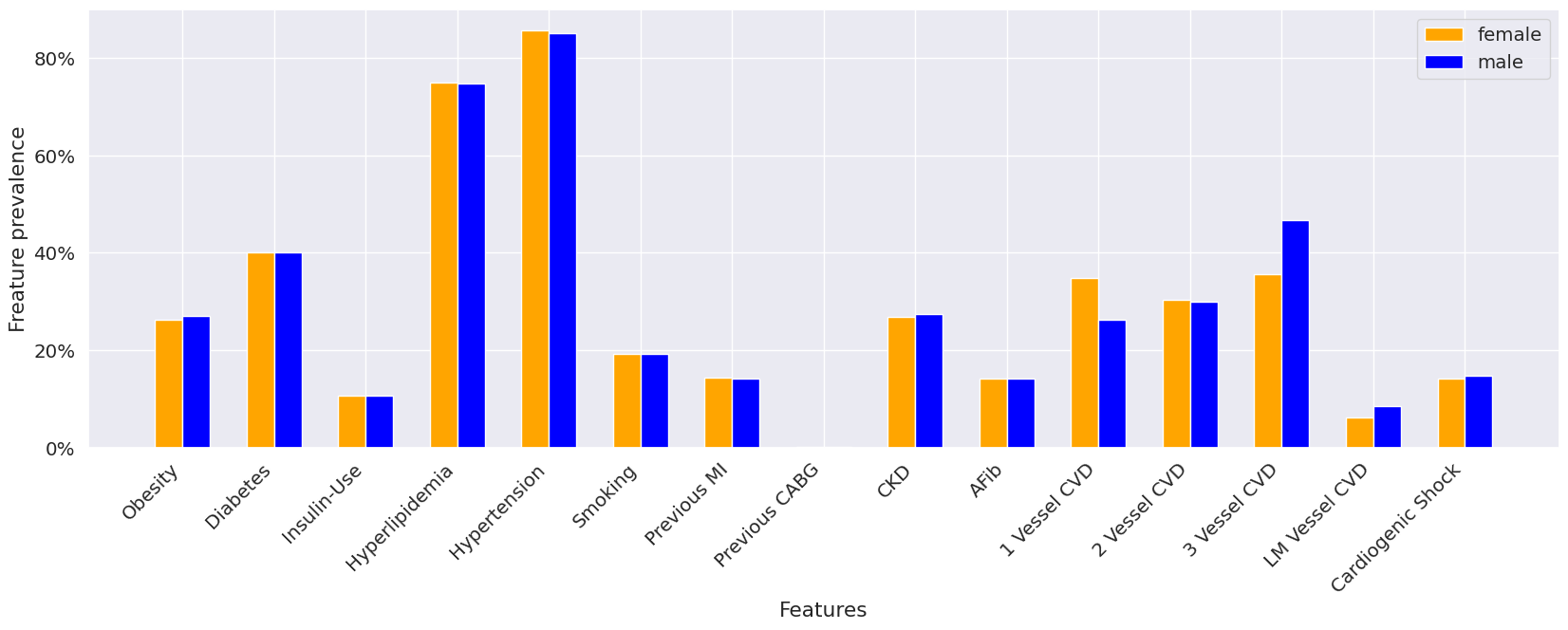


**Supplementary Fig. S11:** Epidemiological and clinical data in the dataset from German health insurance claims after propensity score matching. CKD: chronic kidney disease, AFib: atrial fibrillation, CVD: cardiovascular disease, MI: myocardial infarction, CHF: chronic heart failure, PAD: peripheral artery disease, PCI: percutaneous coronary intervention, CABG: coronary artery bypass graft surgery.

**Supplementary Fig. S12:** Five-year event-free survival from major adverse cardiovascular events (MACE), a composite of myocardial infarction, stroke or death, in the health insurance cohort after propensity score matching. CI=confidence interval; HR=hazard ratio.


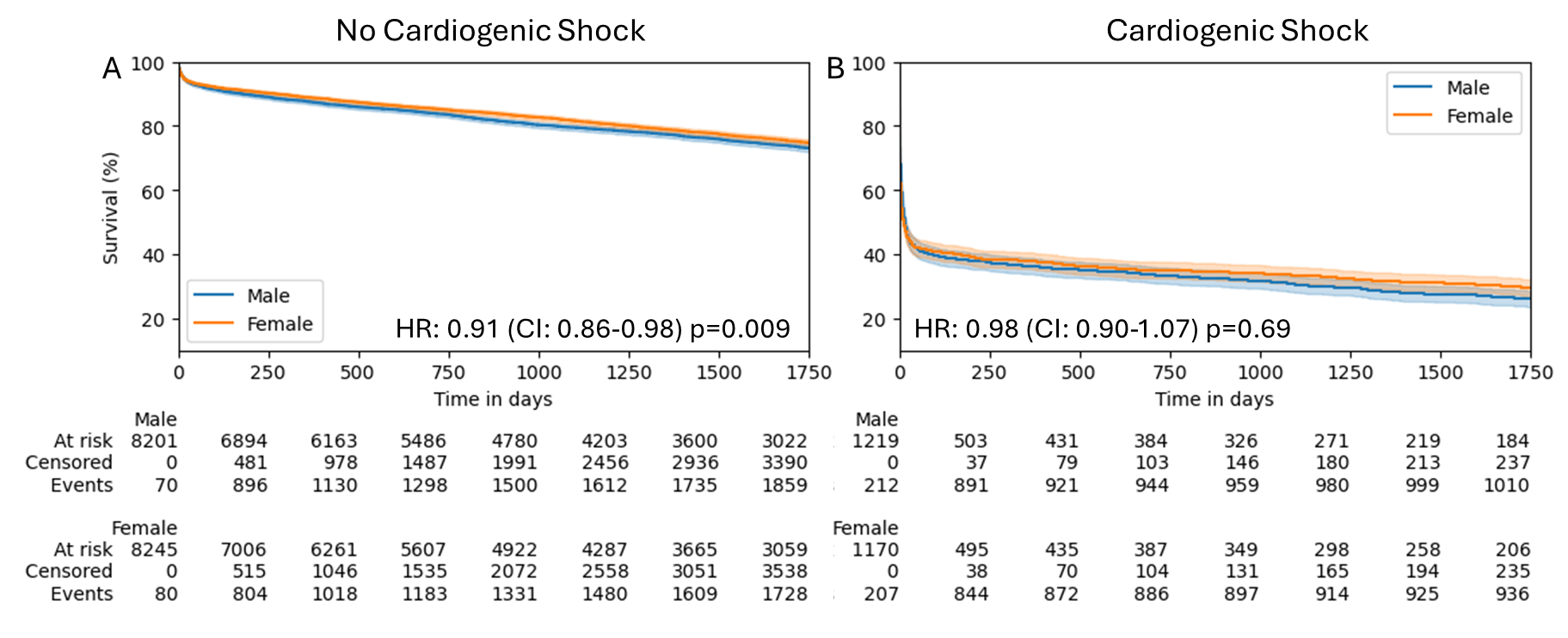


**Supplementary Fig. S13:** **(A)** Without Cardiogenic Shock; **(B)** With Cardiogenic Shock; Kaplan-Meier curves of five-year all-cause mortality between female and male patients in the health insurance cohort after propensity score matching. CI=confidence interval; HR=hazard ratio.


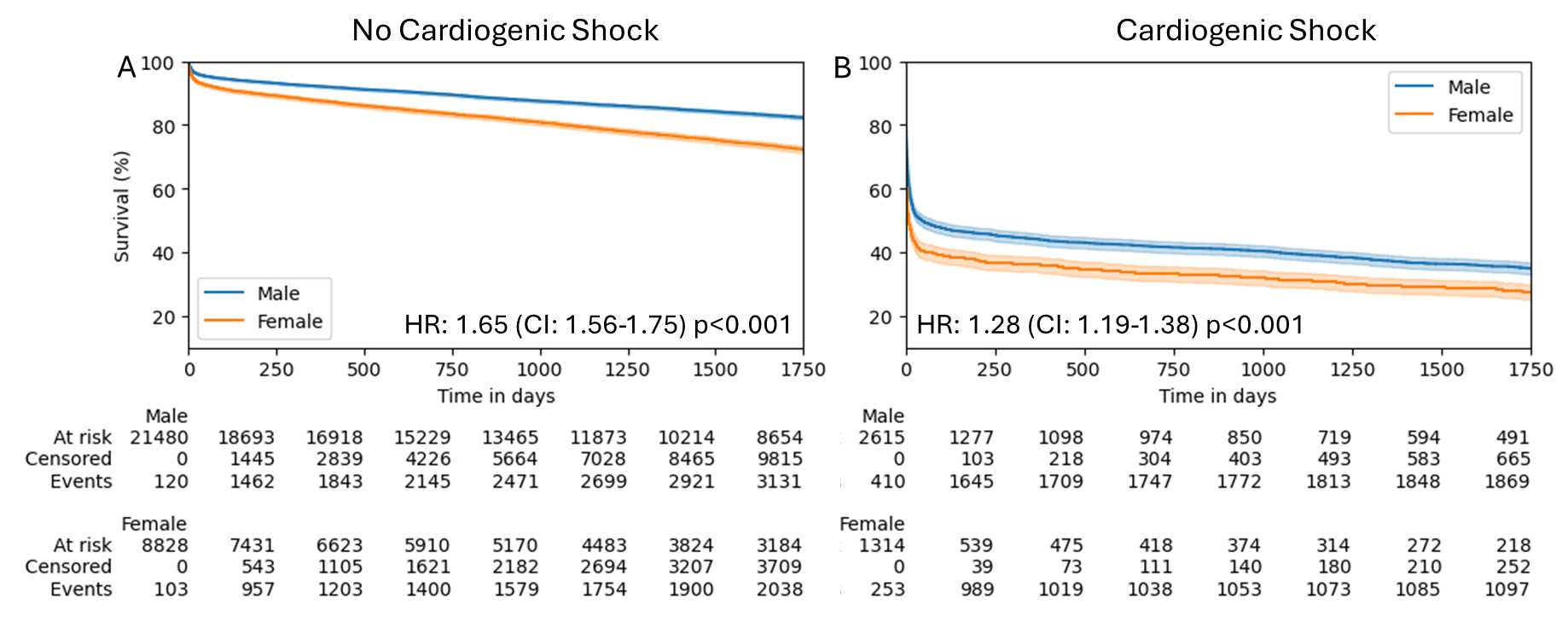


**Supplementary Fig. S14:** **(A)** Without Cardiogenic Shock; **(B)** With Cardiogenic Shock; Kaplan-Meier curves of five-year all-cause mortality between female and male patients in the health insurance cohort before propensity score matching. CI=confidence interval; HR=hazard ratio.

**Supplementary Table S1:** Diagnoses and procedure list.

| **Diagnosis / Procedure / Medication** | **Code** |
| --- | --- |
| ***International Classification of Diseases 10^th^ Revision (ICD-10-GM)*** | |
| Diabetes Mellitus | E10, E11, E12, E13, E14 |
| Diabetes with Endorgan Involvement | E11.2, E11.3, E11.4, E11.5, E10.2, E10.3, E10.4, E10.5 |
| Obesity | E66 |
| Dyslipidemia | E78 |
| Alcohol Abuse | F10 |
| Nicotine Abuse | F17 |
| Transient Ischemic Attack | G45 |
| Arterial Hypertension | I10, I15 |
| Malignant Hypertension | I10.1 |
| Hypertensive Heart and/or Kidney Disease | I11, I12, I13 |
| Unstable Angina Pectoris | I20.0 |
| Stable Angina Pectoris | I20.8 |
| Acute Myocardial Infarction | I21, I22 |
| ST- Segment Elevation Myocardial Infarction | I21.0, I21.1, I21.2, I21.3, I22.0, I22.1, I22.8 |
| Non-ST Elevation Myocardial Infarction | I21.4 |
| Chronic Ischemic Heart Disease | I25 |
| Chronic Ischemic Heart Disease with at least 2 vessel disease | I25.12, I25.13 |
| Atherosclerotic Heart Disease | I25.1 |
| Other Cardiac Arrhythmias | I49 |
| Atrial Fibrillation and Flutter | I48 |
| Chronic Heart Failure | I50 |
| Acute Stroke | I63, I64 |
| Peripheral Artery Disease | I70.2 |
| COPD | J44 |
| Asthma | J45 |
| Chronic Kidney Disease | N18* |
| Chronic Kidney Failure Stage 1 And 2 | N18.1, N18.2 |
| Chronic Kidney Failure Stage 3, 4 And 5 | N18.3, N18.4, N18.5 |
| Chronic Renal Insufficiency Requiring Dialysis | N18.5 |
| Other Chronic Kidney Disease | N18.8, N18.9 |
| Cardiogenic Shock | R57.0 |
| ***German procedure classification system (OPS)*** | |
| (Percutaneous) Transluminal Stenting | 8-837*  Excl.: 8-837.7*, 8-837.8, 8-837.9, 8-837.a*, 8-837.b*, 8-837.c*, 8-837.d*, 8-837.e, 8-837.f, 8-837.g, 8-837.h, 8-837.j, 8-837.s* |
| Coronary Artery Bypass Graft Surgery | 5-36* |
| ***Anatomical Therapeutic Chemical classification system (ATC)*** | |
| Insulin | A10* |

For the German health insurance claims dataset, this study utilized the International Classification of Diseases, 10^th^ Revision, German Modification (ICD-10-GM) codes for diagnoses, the German Procedure Classification System (OPS) codes for medical and surgical procedures, and the Anatomical Therapeutic Chemical (ATC) classification system codes for medications.

**Supplementary Table S2:** Clinical cohort: Baseline characteristics adjusted by propensity score matching.

| 1 | Age |
| --- | --- |
| 2 | Body Mass Index |
| 3 | Hyperlipidemia |
| 4 | Arterial Hypertension |
| 5 | Nicotine Abuse |
| 6 | Chronic Kidney Disease |
| 7 | History of Percutaneous Coronary Intervention |
| 8 | History of Coronary Artery Bypass Grafting |
| 9 | Diabetes Mellitus |
| 10 | Family history of Coronary Artery Disease |

**Supplementary Table S3:** Health insurance claims dataset: Baseline characteristics adjusted by propensity score matching.

| 1 | Age |
| --- | --- |
| 2 | Obesity |
| 3 | Hyperlipidemia |
| 4 | Arterial Hypertension |
| 5 | Nicotine Abuse |
| 6 | Chronic Kidney Disease |
| 7 | Percutaneous Coronary Intervention within the last year |
| 8 | Coronary Artery Bypass Grafting within the last year |
| 9 | Diabetes Mellitus |
| 10 | Insulin intake |
| 11 | Date of myocardial infarction |
| 12 | Cardiogenic shock |
